# Visual attention towards virtual humans as a function of spatial distance and emotional expression: an eye-tracking study

**DOI:** 10.1007/s00426-026-02291-9

**Published:** 2026-05-11

**Authors:** Mariachiara Rapuano, Tina Iachini, Gennaro Ruggiero, Yann Coello

**Affiliations:** 1 Department of Psychology, University of Campania “L. Vanvitelli”, Caserta, Italy; 2 University of Lille, CNRS, UMR 9193 - SCALab - Sciences Cognitives et Sciences Affectives, Lille, France

## Abstract

During social interactions, the way people regulate the space between each other (i.e., the interpersonal distance) reflects the type and quality of the relationship existing between the interactants. Shorter interpersonal distances are for intimate relationships; larger interpersonal distances are for formal social interactions. Thus, we spontaneously choose the appropriate interpersonal distance to feel comfortable and avoid distress. Moreover, when interacting with other people or the environment, the space surrounding the body acts as a protective buffer against potential threats: this space is larger in unsafe situations, smaller in safe situations. Consistently, stimuli entering this area receive close attention and trigger approach or avoidance actions depending on the perceived level of danger. Therefore, one might wonder where people focus their gaze during social interactions to identify and appropriately react to the intentions of others, depending on spatial distance. Using eye-tracking technology in immersive virtual reality, this study sought to address this question by assessing how individuals shift their visual attention to different body parts as a function of the spatial distance (near, middle, far) and facial expression (happy, angry, neutral) of the interactants. Results showed that at near distances the gaze focuses on the faces of the interactant, while at far distances the whole body acquires importance. In the middle space, visual attention is especially drawn to angry interactants, with a focus on action-relevant effectors (i.e., arms). Therefore, the data suggest that spatial distances in social interactions modulate visual attention to specific body parts of the interactant to acquire information useful for preparing appropriate responses, especially when faced with a potential threat: far distances prompt the gathering of information about others (more attention to their whole body); near distances prioritize information about others’ dispositions toward us (more attention to the face); middle distances facilitate preparation for action (more attention to effectors).

## Introduction

In everyday life, people move around their environment and interact with other people. In these dynamics, the space immediately surrounding the body is fundamental. Although we perceive the space around us as a continuum in which interactions are fluid, our brain continuously processes and segments this space into functionally distinct representations (Bogdanova et al., 2021; Coello & Iachini, 2021; Di Pellegrino & Làdavas, 2015; Zanini et al., 2021). As first proposed by Brain (1941), we can distinguish a near space and a far space to indicate a dissociation between a “graspable” distance and a “walking” distance. This notion became popular in the neuro-cognitive domain, with the use of the term peripersonal space (PPS) to refer to the multisensory area around our body where physical interactions with the environment can take place, and the term extrapersonal space (EPS) to refer to the area beyond immediate reach (Brozzoli et al., 2012; Di Pellegrino & Làdavas, 2015; Farnè et al., 2005; Makin et al., 2008; Rizzolatti et al., 1997; Serino, 2019).

In the socio-psychological domain, the surrounding space has also been segmented into different zones depending on the type and quality of human interaction considered. Taking a pioneering approach, Hall (1966) proposed that people typically maintain short social distances during intimate (e.g., with relatives) and personal (e.g., with friends) social interactions, and larger social distances in business-like (e.g., with a supervisor) and public (e.g., addressing a group) situations. More specifically, he divided these distances into Intimate (0–45 cm), Personal (45–120 cm), Social (120–300 cm), and public (300–500 cm). To be noted, Hall (1966) referred to the “senses” to distinguish between the different social distances. Humans’ social distances range from a multisensory close space in which interactants use sight, hearing but also physical touch and smell to interact with each other, to a farther space in which the interactants use mainly sight and hearing to interact with each other (Geers et al., 2024). Interestingly, Personal distance would represent an intermediate zone that accommodates a broader range of relational meanings than those clearly intimate in near space or clearly formal in far space. This range of space is then highly sensitive to socio-emotional modulation: variations in affect, expectations, or context can shift the preferred distance within the typical 45–120 cm range (Iachini et al., 2014, 2016; Ruggiero et al., 2017). Moreover, the short and far social distances described by Hall match quite well the PPS and EPS spatial organisation (Geers & Coello, 2023). However, even the boundary of peripersonal space, typically within the reaching area (about 75 cm), is plastic and modifiable by emotional, social, and environmental factors (e.g., Geers & Coello, 2023; Iachini et al., 2016; Ruggiero et al., 2017).

Since the modulation of these spatial distances occurs mostly during active social encounters, social psychology studies have used the term “interpersonal space” (IPS) to refer to the optimal or preferred distance people spontaneously maintain when interacting with others to ensure a sufficient level of comfort and social appropriateness (Bell & Green, 2005, Hall, 1966; Sommer, 1959). To be noted, IPS represents an emotional zone that people experience as ‘their private space’ and that cannot be intruded upon without causing feelings of discomfort and anxiety (Hall, 1966; Hayduk, 1983; Sommer, 1959). Longstanding research has demonstrated that IPS increases in uncomfortable/threatening situations and decreases in comfortable/safe situations (Gessaroli et al., 2013; Hall, 1966; Hayduk, 1983; Holt et al., 2014; Kennedy et al., 2009; Kennedy & Adolphs, 2014; Lourenco et al., 2011; Tajadura-Jimenez et al., 2011; Ruggiero et al., 2017, 2021). Thus, the IPS represents the preferred distance people use spontaneously for social interactions, but also to protect themselves against potential emotional, psychological or physical threats (Coello & Cartaud, 2021; Felipe & Sommer, 1966; Sommer, 1959).

Taking into account previous literature, here we operationalized distance as three theoretically motivated zones: a near distance, a middle distance and a far distance. The middle distance was included to capture a transitional region where social stimuli may be sufficiently close to signal potential interaction or threat, yet not fully within immediate action range. The intermediate zone may therefore be particularly sensitive to contextual and emotional modulation of attention.

In this light, stimuli entering the near-body space receive particular attention in order to prepare the body to react but also protect the body from potential hazards (Coello & Iachini, 2021). This defensive role makes the near-body space important in organizing individuals’ social interactions and identifying relevant body signals, such as other’s action and dispositions, in order to anticipate others’ intentions and regulate optimal spatial-safety distance accordingly (Gallese et al., 2004; Knutson, 1996; Ruggiero et al., 2017, 2021). Beside verbal cues, individuals can acquire information about others using different sources including facial expressions, body posture and behaviour. The ability to pick up these signals is crucial for quickly forming impressions of others to anticipate their intentions and regulate the optimal safety distance accordingly (Gallese et al., 2004; Knutson, 1996; Ruggiero et al., 2017, 2021). Importantly, individuals give priority to information or cues that can help them to establish whether the other would be beneficial or harmful (Fiske et al., 2007). In this regard, one might wonder where people focus their visual attention (i.e., eye gaze) during social interactions to identify and appropriately react to the intentions of others, depending on the distance.

Emotional signals are among the most important signals during social interactions, and humans use specialised mechanisms to perceive others’ state and intention through their emotional facial expressions (Ekman, 1999; Keltner, Ekman, Gonzaga, & Beer, 2003; Vuilleumier & Pourtois, 2007). Prior research has demonstrated that emotional stimuli, particularly those with negative content, are able to quickly attract and retain attention (e.g., Carter & Luke, 2020; Knickerbocker et al., 2019; Scott et al., 2012; Stephenson et al., 2019). In particular, angry and fearful cues from the face and the body attract more attention than happy cues (e.g., Kret et al., 2013; Morris et al., 1997, 1998) and produce more physiological responses (Cartaud et al., 2020; Kennedy et al., 2009; Ruggiero et al., 2021). Supposedly, negative-aggressive signals are perceived as a more direct threat to physical harm (de Gelder et al., 2010; Kret et al., 2013; Rapuano et al., 2023). When observing an individual expressing anger, several processes are prompted: first, the attention is drawn to the threat, especially to the face of the individual (Fox & Damjanovic, 2006; Green et al., 2003; Lundqvist & Ohman, 2005; Morris et al., 1997, 1998) and then to his/her body (Bannerman et al., 2009; Kret et al., 2013); second, arousal levels increase to facilitate fast behavioural reactions (e.g., our heart rate changes, Bradley et al., 2006; Ruggiero et al., 2021).

However, these processes are affected by the distance between the social stimulus and our body. Indeed, stimuli closer to the body can be perceived as more threatening and cause an increase in defensive responses (Cooke & Graziano, 2003; Iachini et al., 2017; Kennedy et al., 2009; Sambo & Iannetti, 2013; Vagnoni et al., 2015). Consistently, we tend to keep individuals with an angry facial expression at a larger distance than happy ones (Ruggiero et al., 2017, 2021). Instead, stimuli in far space are less relevant for immediate actions and less “threatening” for the integrity of the body (Coello & Cartaud, 2021).

According to de Vignemont and Iannetti (2015; but see also Serino, 2019; Zanini et al., 2021), the attention to specific body parts could represent a viable way to study the putative near-body space representations. In fact, the authors suggested that attention should be focused on the hands because they are crucial for goal-directed actions, while it should be spread throughout the body and specifically on the face to prompt defensive avoidance actions. This raises the question of what happens when we face a potential social threat. More specifically, how long is visual attention allocated to specific body parts depending on the distance of the social stimulus and its emotional expression?

The current study used eye-tracking in immersive virtual reality to assess how we allocate visual attention on different body-parts depending on the distance of the social stimulus (near, middle, and far) and its emotional expression (angry, neutral, happy). To this aim, participants in the present study were asked to judge if the distance between themselves and a virtual human was sufficient for interacting (e.g., to talk/listen efficiently). They had to provide ‘yes - no’ responses while interacting with virtual humans presented at different distances ranging from the near to the far space while eye-tracking measures were recorded. Eye-tracking technology was chosen because it is an effective method for studying attentional, orientation, affective and unconscious processes towards different visual stimuli (e.g., Carter & Luke, 2020). We expected that, when interacting with unfamiliar individuals at close range, attention would primarily be directed towards their face rather than their whole body, as facial expressions provide crucial emotional cues for inferring intentions. At greater distances, however, gaze may shift toward action-relevant effectors and on the body as a whole to assess others’ action possibilities. These predictions, however, are likely to be modulated by the emotional significance of facial expressions.

## Materials and methods

### Participants

The sample size was determined using G*power 3.1.9.2 software (Faul et al., 2009). With α = 0.05, power (1-β) = 0.95, one group of participants and five repeated measures (average correlations among measures = 0.50), the minimum required sample size was 32 participants to detect an effect size (Cohen’s f) = 0.25. For the current study, 35 volunteers (20 females) aged 20–29 (M = 23.9; sd = 3) were included. Participants were naïve as to the experimental hypotheses, had normal vision, and reported no history of neurological or psychiatric disorders. Given that individuals with high anxiety tend to overreact to social or emotional stimuli, only participants with low anxiety levels (e.g., STAI-Trait scores < 40; Spielberger et al., 1983) were included. All participants provided written informed consent to participate in the experiment, which was approved by the Ethical Committee of the University of Campania L. Vanvitelli (#6/2025).

### Setting and apparatus

The study was conducted at the Laboratory of Cognitive Science and Immersive Virtual Reality (CS-IVR, Department of Psychology). The experimental room was set with the immersive virtual reality (IVR) equipment consisting in two cameras connected with the Head-Mounted Display (HMD) “HTC Vive Pro Eye” (HTC, Corporation, Taipei, Taiwan) having two OLED display panels with a resolution of 1440 × 1600 per eye (global resolution 2880 × 1600), a refresh rate of 90 Hz and a 110-degree field of view, integrated with the Tobii Eye-tracking Retrofit system (Tobii Technology, Stockholm, Sweden, https://www.tobii.com/); a workstation supported the Unity real-time 3D development platform (Unity technology) that was used to visualize the virtual scenario and the virtual stimuli.

### Virtual scenario and virtual stimuli

**Fig. 1:**
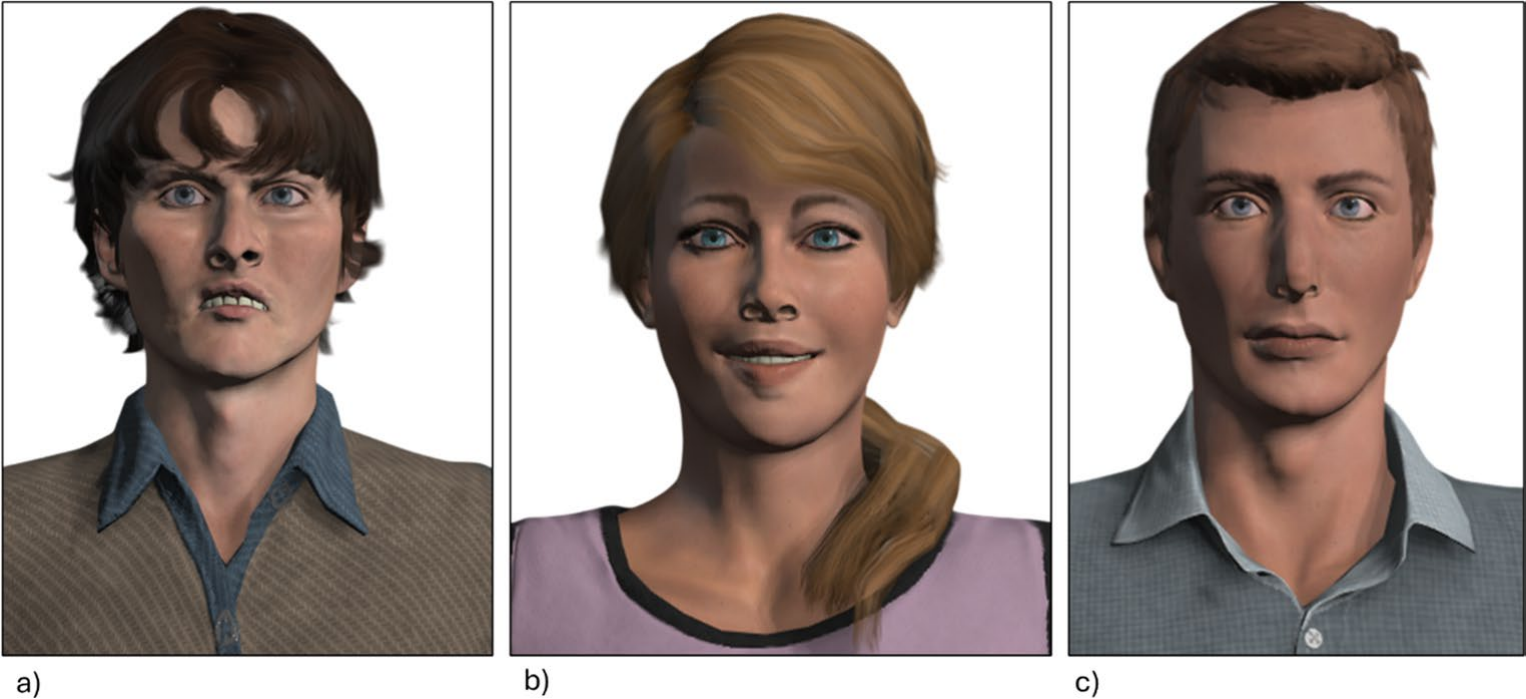
The panel represents examples of experimental stimuli selected for the study. Panel (**a**) shows a male virtual human with an angry facial expression; panel (**b**) shows a female virtual human with a happy facial expression; panel (**c**) shows a male virtual human with a neutral facial expression

The virtual environment, created with Unity (Unity technologies), depicted a spacious room with grey walls and a light-coloured floor. Virtual stimuli consisted of male and female young adults exhibiting a happy, angry, or neutral facial expression. Facial expressions were obtained by modelling the virtual humans’ faces using Adobe Fuse CC (Fuse Character Creator) and a 3D computer graphics software, following the KDEF free data-base (Karolinska Directed Emotional Faces, Lundqvist et al., 1998). All virtual humans wore casual clothes and were perceived as real persons. Their gaze was kept looking straight ahead throughout the trials (Bailenson et al., 2003). Eleven subjects (not included in the study) rated on a 9-point scale how much the faces presented on a PC screen appeared happy, angry, annoying (unpleasant), quiet (pleasant). The selection of stimuli was based on the virtual humans’ capacity to consistently evoke the intended emotion (high ratings of happiness for happy facial expressions, high ratings of anger for angry facial expressions) and, at the same time, not to significantly elicit the opposite emotion. This criterion allowed us to identify stimuli with greater expressive clarity and emotional specificity, ensuring that facial expressions were interpreted by participants in a manner consistent with the experimental conditions (see Table 1). Following this evaluation, 36 virtual humans were selected (see Fig. 1).

**Table 1:** Mean and standard deviation (s.d.) of virtual humans’ ratings

	Happiness		Anger		Quite/ pleasant		Annoying/ unpleasant	
	Mean	s.d.	Mean	s.d.	Mean	s.d.	Mean	s.d.
Happy virtual humans	6.38	1.56	1.27	0.64	6.36	0.44	1.81	1.25
Angry virtual humans	1.23	0.51	7.27	1.63	1.73	0.96	7.33	0.27
Neutral virtual humans	2.80	0.44	2.56	0.71	4.76	0.53	2.80	0.82

### Procedure

Upon entering the experimental room, participants provided written informed consent and completed the State-Trait Anxiety Inventory. They then received instructions about the experimental procedure and were invited to wear the HMD to freely explore the virtual room using their gaze. Afterwards, they were given a key-press device to hold in their dominant hand. Once participants were familiarized with the IVR setup, the experimenter initiated the experimental session.

Participants then completed the social distance judgment task, which required them to assess whether the distance between themselves and a virtual human was appropriate for social interaction. Specifically, the instruction was: *‘You will see a person standing still at a certain distance from you. As soon as the person disappears*,* please press the right (or left) key if you think the distance between you and the other person is sufficient to interact with him/her; press the left (or right) key if you think the distance is not sufficient.’* The mapping of response keys was counterbalanced across participants to control for response biases.

The experimental sequence consisted of task instructions (10 s), followed by a fixation cross presented for 300 milliseconds. Subsequently, a virtual human appeared at a specific distance for a duration of 3 s. Although the virtual humans remained static, their posture simulated a walking motion, characterized by the typical arm and leg swing. Once the stimulus disappeared, participants provided their response, after which the next trial began. Virtual humans were presented at nine different distances: three in near space (45 cm, 60 cm, 75 cm), three in middle space (90 cm, 105 cm, 120 cm), and three in far space (135 cm, 150 cm, 165 cm). Throughout the experimental session, participants stood still and saw each happy, angry, and neutral virtual human three times, once per distance category, for a total of 108 trials. The presentation of the 36 virtual humans was counterbalanced across participants, such that each virtual human appeared at each of the nine distances across the sample.

At the beginning of the experimental procedure, participants received a four-trial training session to become familiar with the task and the IVR environment. Midway through the experiment, a 5-minute break was provided, during which participants removed the HMD. At the end of the experimental session, participants were asked to report their impressions of the virtual humans. They indicated they could clearly identify the facial expressions as if they were realistic persons. Finally, participants completed the State-Trait Anxiety Inventory.

### Data processing

We defined three primary regions of interest (ROIs) on the virtual humans: the face, the body (i.e., torso plus legs), and the arms (i.e., arms plus hands), to investigate the contribution of global body parts in attracting visual attention during social interactions. Additionally, to explore the role of specific body parts (e.g., action-relevant effectors such as the hands), we further subdivided the ROIs to include the upper body and legs, as distinct regions. Gaze duration (in seconds) was used as the main dependent variable. This measure reflects the proportion of time participants spent fixating on each ROI relative to the total exposure duration (i.e., 3 s per trial).

### Data analysis

In each trial, gaze duration (i.e., the proportion of time spent fixating on each ROI) was recorded. Subsequently, mean gaze durations were computed for each combination of emotional expression, spatial distance, and ROI. To simplify the analysis, the nine original viewing distances were grouped into three categories: near (45 cm, 60 cm, 75 cm), middle (90 cm, 105 cm, 120 cm), and far (135 cm, 150 cm, 165 cm), by averaging the gaze durations across the three steps within each distance category. A 3 × 3 × 3 repeated-measures ANOVA was conducted on gaze duration, with Facial Expression (3 levels: Happy, Angry, Neutral), Distance (3 levels: Near, Middle, Far), and ROI (3 levels: Face, Body, Arms) as within-subject factors. This analysis aimed to assess how emotional expression, interpersonal distance, and body region jointly influenced visual attention during the social distance judgment task.

To further investigate the role of specific body parts (i.e., upper body and legs) in attracting selective attention, an additional repeated-measures ANOVA was conducted. The analysis included three within-subject factors: Facial Expression (3 levels: Happy, Angry, Neutral), Distance (3 levels: Near, Middle, Far), and ROI (2 levels: Upper Body vs. Legs).

To check whether ROI size variation due to distance manipulation affected the results, we conducted a regression analysis using the nine physical Distances as predictors and as dependent variable the fixation time on each ROI of the 3 body parts (Face, Body, Arms) for each Emotion.

Finally, we analysed the subjective judgments of the appropriateness of distances for social interaction. A 3 × 3 repeated-measures ANOVA with Facial Expression (Happy, Angry, Neutral) and Distance (Near, Middle, Far) as within-subject factors was performed on mean judgments for each combination of facial expression and distance.

Data points falling outside the range of M ± 2.5 SD (representing 2.8% of the dataset) were excluded from the analysis. Post hoc comparisons were performed using Bonferroni correction, and effect sizes for significant results were reported using partial eta-squared (η_p_²). Only statistically significant effects are reported.

## Results

### Overall body parts

Statistical analysis revealed a main effect of Facial expression (F(2,68) = 31.7, *p* <.00001, η_p_² = 0.48). Gaze duration was longer on angry (M = 0.347, SD= 0.27) than happy (M = 0.332, SD= 0.28) and neutral (M = 0.331, SD = 0.28) virtual humans (at least, *p* <.00001).

A main effect of Distance was also found (F(2,68) = 31.2, *p* <.00001, η_p_²= 0.48), with gaze duration being longer on the stimuli presented in the middle (M = 0.347; SD = 0.27) than the near (M = 0.331, SD= 0.30) and far (M = 0.333, SD = 0.27) spaces (at least, *p* <.000001).

Statistical analysis also showed a main effect of ROI (F(2,68) = 120.8, *p* <.00001, η_p_² = 0.78). Gaze duration was longer on faces (M = 0.627; SD= 0.18) than body (M = 0.334; SD = 0.17) and arms (M = 0.050; SD= 0.06; at least *p* <.00001). Moreover, gaze duration was longer for the body than arms (*p* <.00001).

Facial expression and Distance significantly interacted (F(4,136) = 44.7, *p* <.00001, η_p_² = 0.57); this was due to angry virtual humans attracting more visual attention in the middle space than other conditions (*p* <.00001).

The interaction between Facial expression and ROI was also significant (F(4,136) = 10.9, *p* <.00001, η_p_² = 0.24). Pairwise comparisons revealed that gaze duration was longer for face than body and arms, and longer for body than arms in all emotion conditions (at least, *p* <.00001). Furthermore, the arms of angry virtual humans attracted the gaze longer than the arms of happy and neutral virtual humans (at least, *p* <.00001).

A significant interaction between Distance and ROI was also found (F(4,136) = 31.3, *p* <.00001, η_p_² = 0.50). Although the gaze duration was longer on the faces than the body and arms at all distances, the effect was significantly more marked in near space than in all other combinations (at least, *p* <.05). Gaze duration was also longer on the body than the arms at all distances (at least, *p* <.00001), but the effect was stronger in far space than in middle and near spaces (at least, *p* <.01).

**Fig. 2:**
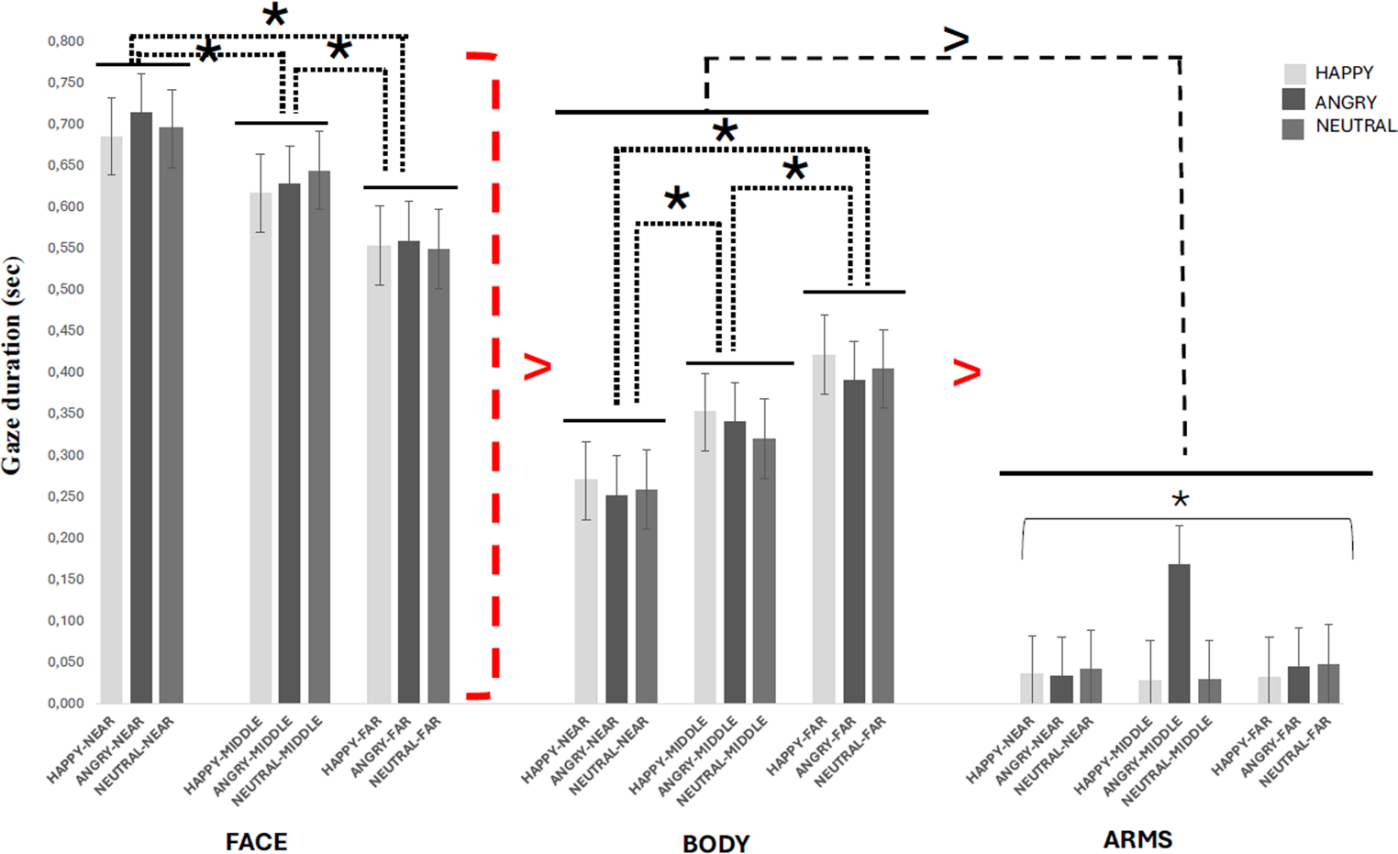
The graph shows the gaze duration as a function of virtual humans’ facial expression (happy, angry, neutral), spatial distance (near, middle, far) and ROI (face, body, arms). The solid black lines indicate which experimental conditions differ from the others. The red dashed line with the red major mark indicates the experimental conditions that are significantly different from all others; the black dashed line with the black major mark connect experimental conditions that are significantly different from other experimental conditions. Finally, the black dashed lines with black asterisks indicate the experimental conditions that differ significantly from each other. The error bars represent the standard error. Asterisks indicate significant differences

A three-way interaction emerged F(8,272) = 10.1, *p* <.00001, η_p_² = 0.23). Post-hoc analysis revealed that gaze duration was longer on the faces than the body and arms, especially in near space compared to middle and far spaces, and in middle space compared to far space, regardless of the emotion condition (at least, *p* <.05). The body attracted more visual attention than the arms of virtual humans and more in far space than in middle and near spaces, and more in middle than near spaces, regardless of the emotion condition (at least, *p* <.05). As for the arms of virtual humans, the post-hoc analysis revealed that the gaze duration on the arms was longer with angry virtual humans in middle space than with happy and neutral virtual humans in all distance conditions (at least, *p* <.0001; see Fig. 2).

To check the robustness of the results, we also analysed the raw data using the same ANOVA model as above. The general pattern of main and interaction effects, as well as post-hoc comparisons, was confirmed. We also checked whether the results were influenced by the gender of the virtual humans and participants. We added the factor between Participant Gender and the factor within Virtual Human Gender to the above analysis model. The results showed no significant effects, and therefore the analysis was not reported.

### Specific body parts

#### Upper body vs. legs

Statistical analysis revealed a main effect of Distance (F(2,68) = 24.91, *p* <.000001, η_p_² = 0.42). Gaze duration was longer in the far (M = 0.156; SD = 0.13) than the middle (M = 0.135; SD = 0.12) and near (M = 0.111; SD = 0.11) spaces (at least, *p* <.01).

A main effect of ROI was also observed (F(1,34) = 57.62, *p* <.00001, η_p_² = 0.63), with gaze duration being longer on the upper body part (M=0.212; SD = 0.12) than the legs (M = 0.057; SD = 0.06).

**Fig. 3:**
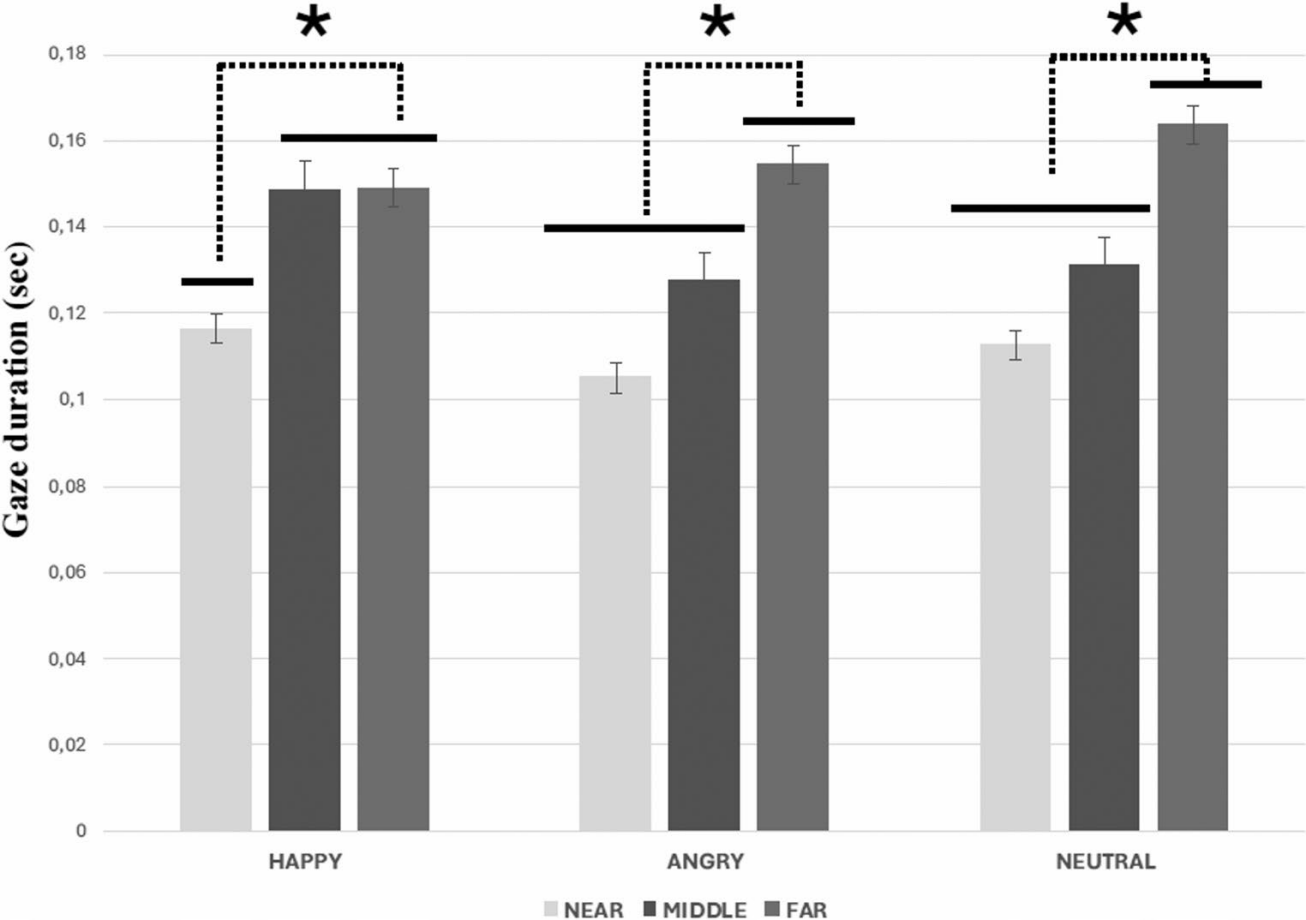
The graph shows the gaze duration as a function of virtual humans’ facial expression (happy-angry-neutral) and spatial distance (near-middle-far). The solid black lines indicate which experimental conditions differ from the others. The black dashed lines with black asterisks connect the experimental conditions that significantly differ from each other. The error bars represent the standard error

Facial expression and Distance significantly interacted (F(4,136) = 2.86, *p* <.05, η_p_² = 0.08). Post-hoc analysis showed that with angry and neutral virtual humans, gaze duration was longer in the far space compared to the middle and near spaces (at least, *p* <.05), and with happy virtual humans gaze duration was longer in the far and middle spaces compared to the near one (at least, *p* <.01; see Fig. 3).

#### Regression analysis

The analysis revealed that distances had an opposite effect on Face and Body. More specifically, a significant negative effect emerged with Angry (t = −12.829, *p* <.0001, β = − 0.979, SE = 0.076), Happy (t = −11.291, *p* <.0001, β = − 0.974, SE = 0.086) and Neutral (t = −11.734, *p* <.0001, β = − 0.976, SE = 0.083) Faces, indicating that fixation time decreased with increasing distance. A significant positive effect emerged with Angry (t = 4.585, *p* <.005, β = 0.866, SE = 0.189), Happy (t = 5.230, *p* <.005, β = 0.892, SE = 0.171) and Neutral (t = 10.732, *p* <.0001, β = 0.971, SE = 0.090) Bodies, indicating that fixation time increased with increasing distance. In sharp contrast, no significant effect was found with Arm.

#### Subjective judgments of the appropriateness of distances

ANOVA revealed a main effect of Facial expression (F(2,70) = 21.27, *p* <.00001, η_p_² = 0.38). The post-hoc analysis showed that the appropriateness of distances for social interactions was judged lower in the presence of angry (M = 0.536, SD= 0.387) than happy (M = 0.732, SD= 0.311) and neutral (M = 0.674, SD= 0.342) faces (at least *p* <.0001).

Facial expression and Distance significantly interacted (F(4,140) = 3.71, *p* <.005, η_p_²= 0.11). With happy and neutral faces, far distances were evaluated as less appropriate than near and intermediate distances (at least *p* <.0001). With angry faces, intermediate distances were considered as more appropriate than far ones (*p* <.005). Near and Intermediate distances were considered more appropriate with happy and neutral faces than angry faces (at least *p* <.0001).

## Discussion

The present study aimed to investigate how observers allocate visual attention to different body regions of unfamiliar individuals during social interactions occurring at varying spatial distances in a virtual environment. Participants performed a social-distance judgment task with virtual humans displaying happy, angry, or neutral facial expressions, presented at near, middle, and far distances. We hypothesized that gaze duration—used as an index of visual attention—would be modulated both by the interpersonal distance and by the emotional expressiveness of the virtual interactants.

Before continuing, it is important to address an important criticism. Since the ROIs defined for the virtual humans at farther distances should be smaller, one can argue that the results may just reflect the ROI size difference by physical distance. As a consequence, if ROI size alone determined fixation time, then we should expect a uniform decrease in fixation duration across all ROIs as distance increased. Instead, results showed an opposite effect of distance on fixation time: a negative effect with faces (fixation decreased with increasing distance) combined with all emotions; a positive effect with bodies (fixation increased with increasing distance) combined with all emotions. No significant effect with arms appeared. This pattern is incompatible with a simple size-driven explanation since fixation time shifted systematically depending on body parts.

Consistent with our hypotheses, the results revealed that at near distances, visual attention was predominantly focused on the face, whereas at farther distances, gaze was distributed more broadly across the entire body.

Importantly, considering the analysis on overall body parts, the middle spatial zone appeared to be particularly sensitive to approaching stimuli: visual attention was significantly longer on stimuli presented in this region compared to those in the near and far spaces, especially when the virtual humans expressed anger.

Consistent with our hypotheses, specific body parts such as the upper body and legs attracted more attention at far distances, while the arms garnered more attention in the middle space—particularly in response to angry virtual humans. These findings support the notion of heightened sensitivity to stimuli entering this spatial zone (Bufacchi & Iannetti, 2018; Coello & Iachini, 2021; de Vignemont & Farnè, 2024; Di Pellegrino & Làvadas, 2015).

The results about the subjective judgements of the appropriateness of distances for social interaction give further support to the idea that spatial distance carries emotional and social meaning. Indeed, distances were judged as globally less appropriate in the presence of angry than happy and neutral facial expressions. More specifically, in the presence of virtual humans showing happy and neutral facial expressions, far distances were evaluated as less appropriate than near and intermediate distances. Instead, with angry faces, intermediate distances were considered as more appropriate than far ones. Near and Intermediate distances were considered more appropriate with happy and neutral faces than angry faces.

Two main observations emerge from the present data. First, during interactions with virtual humans, the near space is critical for gathering information about others’ emotional states and their potential dispositions—whether helpful or threatening (e.g., Cartaud et al., 2018; Ruggiero et al., 2017, 2021). At farther distances, the body as a whole gains importance, allowing for the evaluation of others’ actions or intentions (e.g., Thorat & Peelen, 2022). Second, the middle space appears to be a particularly salient zone for social interaction. Social stimuli entering this area receive enhanced attentional processing, especially when conveying negative emotions. This finding aligns with previous research showing that negative stimuli (such as angry or fearful cues) capture more attention than positive ones (e.g., Kret et al., 2013; Morris et al., 1997, 1998; Rapuano et al., 2023). Moreover, it is consistent with the defensive role of peripersonal space, the multisensory near-body area that functions as a protective buffer by triggering defensive or avoidance behaviours (Coello et al., 2012; de Vignemont & Farnè, 2024; Di Pellegrino & Làdavas, 2015; Graziano & Cooke, 2006).

Importantly, the present study demonstrated that body effectors relevant for protective or action-oriented responses, such as the arms, captured individuals’ visual attention particularly in the middle space when interacting with angry virtual humans. This is likely because this spatial zone is critical for rapidly assessing whether the other poses a threat, enabling timely and appropriate reactions. In fact, this area can be conceptualized as a ‘flight zone’, i.e., a buffer region that, when breached, triggers an individual’s defensive or retreat responses (Felipe & Sommer, 1966; Graziano & Cooke, 2006; Graziano, 2017; Hediger, 1955). This zone may represent a safety space triggering avoidance behaviour following intrusion, which is essential for self-protection (Hayduk, 1983; Dosey & Meisels, 1969; Siegman & Feldstein, 2014). From a functional perspective, reacting too late (when the other is already too close) or too early (when the other is still far away) would be maladaptive, highlighting the importance of this intermediate spatial boundary.

Notably, in the presence of a potential threat, participants’ gaze allocation was enhanced toward the arms of the virtual human. This likely reflects the need to gather additional information about the interactant’s possible intention, especially as negative facial expressions signal imminent danger, requiring sustained attentional engagement relative to their proximity to the observer’s body (Hediger, 1955; Keltner et al., 2003; Ruggiero et al., 2017, 2021; Coello & Cartaud, 2021). This observation aligns with prior research highlighting the importance of arms (and hands) in expressing and recognizing emotions conveyed through body language, particularly in the context of threat-related emotions (e.g., Ross & Flack, 2020; Witkower & Tracy, 2018; see also Calbi et al., 2021).

To conclude, the present eye-tracking results suggest that, during social interactions, visual attention is preferentially allocated to bodily cues signalling potential threat or action tendencies as a function of interpersonal distance (e.g., Green et al., 2003; Kret et al., 2013; Schrammel et al., 2009). More specifically, within near space, facial processing may predominate because social and emotional evaluation becomes urgent and immediate. Within middle space, the stimulus is sufficiently close to signal a potential threat, yet not within immediate interaction range, which may enhance the monitoring of action-relevant effectors. Within far space, threat imminence is reduced, and defensive preparation becomes less urgent. More specifically, in near space, facial processing may prevail because social/emotional evaluation becomes urgent and immediate. In middle space, the stimulus is close enough to signal a potential threat, but not yet within the range of immediate interaction, possibly enhancing the monitoring of effectors relevant to action. In the far space, the imminence of the threat is reduced and defence preparation is less urgent. Importantly, in line with the perspective proposed by de Vignemont and Farnè (2024), these preliminary findings support the notion that the primary function of near-body space representations is protective. Attention is not only distributed across one’s own body but also selectively focused on the whole body, face, and effectors of others to anticipate and prepare appropriate responses to potential threats (i.e., Iachini et al., 2017).

Furthermore, spatial distances shape the shift of visual attention making available a range of social information about others: far distances allow for gathering general information about others through attention to their whole body; near distances prioritize information about others’ dispositions toward us, with attention focused on the face; and middle distances facilitate the preparation of action by directing attention to action-relevant effectors. Notably, this is also coherent with evidence about the effect of spatial distances on the impression we form of other persons in social situations. For example, people perceived an interactant to be more dominant as he/she stands closer (e.g., Patterson & Sechrest, 1970; Trifonova et al., 2024).

### Future research directions

Although only participants with low levels of anxiety were considered for this study, we should investigate whether and to what extent anxiety levels could be related to the way visual attention shifts to virtual humans with different facial expressions and at different spatial distances from the interlocutor. The reason lies in the fact that there is evidence in the literature about the influence of anxiety on how we adjust interpersonal distance during social exchanges with virtual humans (e.g., Iachini et al., 2015). Otherwise, no significant results were observed here. A further study could be considered to probe this aspect, with a particular focus on social anxiety. But, even more, the findings we presented pave the way for future research that should explore the effect of the factors studied here on individual differences or on populations with impaired social behaviours, starting with standardised questionnaires and tests. Finally, fixation path data is needed to show how visual attention shifts across the ROIs. This would provide a reliable index of body part prioritisation by reconstructing the temporal sequence of gaze allocation.

## Data Availability

The data presented in this study are available on request from the corresponding author.
